# What are the challenges midwives face working with refugee women and how can they be addressed?

**DOI:** 10.18332/ejm/168942

**Published:** 2023-08-04

**Authors:** Elena Triantafyllou, Victoria Vivilaki

**Affiliations:** 1 Department of Midwifery, School of Health and Care Sciences, University of West Attica, Athens, Greece

**Keywords:** midwives experiences, refugee mothers, migrant mothers, barriers, perinatal care

With the numbers of refugees gradually increasing over the years^1^, the challenges faced by midwives working with refugees are various and complex. Midwives are required to provide care in difficult, limited resourced environments, frequently working with women who have experienced traumatic events, speak different languages, and come from various cultural backgrounds. For these challenges to be addressed, a multi-faceted approach with the involvement of policymakers, healthcare organizations, and the midwives is required.

The role of the midwives in ensuring safe childbirth and maternal health is vital. However, one of the most significant obstacles they face is the limited resources and lack of infrastructure to provide the necessary care. Women that give birth in refugee camps are at higher risk of complications, including infection, hemorrhage, and obstructed labor, due to limited access to skilled healthcare professionals and basic medical supplies and equipment^2^.

Another challenge is the absence of specialized training for midwives working with refugees. Refugees frequently originate from regions with a high incidence of contagious illnesses and could have been subjected to traumatic experiences and violence. Midwives working with these women must have specialized training in the unique health challenges faced by refugees, including the provision of mental health support for trauma survivors and navigation of cultural differences that may impact the provision of healthcare. Midwives must be sensitive to these variations and work collaboratively with women to establish culturally appropriate care plans^3-5^.

The language barrier is another significant obstacle faced by midwives providing care to refugee women. Refugee women may not speak the same language as the midwives, which can lead to miscommunication resulting in errors in treatment that can cause adverse outcomes for the health of the mother and child^5^.

To conclude, midwives face numerous challenges when providing care to refugee women. These challenges include inadequate resources, lack of specialized training, and language barriers. It is essential for policymakers and healthcare organizations to make the health needs of refugees a priority and enable the provision of the necessary resources and training to midwives working with refugee women. Only by addressing these challenges can we ensure that not only all women, regardless of their circumstances, receive the maternal healthcare they need and deserve, but also that midwives are working in safe and enabling environments, to their full scope of practice^6^.

## Data Availability

Data sharing is not applicable to this article as no new data were created.
